# Community health needs assessment with precede-proceed model: a mixed methods study

**DOI:** 10.1186/1472-6963-9-181

**Published:** 2009-10-09

**Authors:** Ying Li, Jia Cao, Hui Lin, Daikun Li, Yang Wang, Jia He

**Affiliations:** 1 Department of Social Medicine and Health Service Management, College of Military Preventive Medicine, Third Military Medical University, No.30 Gaotanyan Road, Shapingba district, Chongqing, PR China; 2 College of Military Preventive Medicine, Third Military Medical University, No.30 Gaotanyan Road, Shapingba district, Chongqing, PR China; 3 Department of Epidemiology, College of Military Preventive Medicine, Third Military Medical University, No.30 Gaotanyan Road, Shapingba District, Chongqing, PR China; 4 Department of Laboratory Medicine, Southwest Hospital, Third Military Medical University, No.30 Gaotanyan Road, Shapingba district, Chongqing, PR China; 5 School of Public Health, Chongqing Medical University, No.1 Yixueyuan Road, Chongqing, PR China

## Abstract

**Background:**

Community health services in China have developed over the last few decades. In order to use limited health resources more effectively, we conducted a community health needs assessment. This aimed to provide an understanding of the community's health problems and the range of potential factors affecting risk behaviours for the priority health problems.

**Methods:**

We used the precede-proceed model for the needs assessment. Triangulation of data, methods and researchers were employed in data collection.

**Results:**

Main findings include: cardiovascular diseases (CVDs) were identified as the priority health problems in the study communities; risk factors associated with CVDs included smoking, physical inactivity and unhealthy eating behaviours, particularly amongst male residents with low education level; factors negatively affecting behaviours were classified into predisposing factors (limited knowledge, beliefs and lack of perceived needs), enabling factors (limited access to health promotion activities, unawareness of health promotion, lack of work-site and school health promotion, absence of health promotion related policy) and reinforcing factors (culture). Policies and organization were not perfect; there were limited staff skilled in providing health promotion in the community.

**Conclusion:**

CVDs were identified by the communities as priority health problems. Future health programs should focus on smoking, physical inactivity and unhealthy eating behaviours. Behaviour change strategies should take predisposing factors, enabling factors and reinforcing factors into consideration. Policies, organization and human resource need strengthening.

## Background

Health services within the community provide people with a more convenient and quicker medical service and ease the pressure on large hospitals. In the Chinese health sector reform, the government has prioritized improving community health services in an attempt to discourage people from using hospital services for minor illnesses [1]. Several problems exist in the urban health system, for example, health resources were allocated to different levels of health facilities with little going to community health services, resulting in under development of these services in both preventive and curative health care. In response to these problems, the Chinese government drew up policies on community health service development between1997 and 2006 [2-5]. These policies emphasized the important role of community health service in urban public health and the medical service system, and provided guidance for the Chinese community health service reform. In 2006, the Chinese Premier at the National People's Congress (NPC) emphasized the government's role in developing community health services [1]. A new community-based urban medical service system is set to be established in the next five years by re-allocating resources, raising funds and enhancing the training of staff [1]. Community Health Centers (CHCs) are considered an important part of the Chinese Urban Health Reform System. In 2002, 31 provinces including the autonomous regions and central government-ruled cities such as Beijing, Shanghai, ChongQing and Tianjin had a total of around 2406 CHCs and 9700 health service stations [6] and in 2009 the Ministry of Health (MOH) estimated that there are more than 7200 CHCs and 22,000 community health stations in China [7]. The CHCs are involved in delivering six main functions: disease prevention and control, health care services, health education, family planning, medical treatment service and community rehabilitation [6]. The Chinese government has recently attached great importance to community based health promotion. In order to further strengthen community based health education and health promotion, the MOH has developed a programme for community based health education and health promotion [8]. Local government has sponsored community based health promotion programs and activities throughout China [9,10]. Although there are many models for health promotion, studies have shown that the PRECEDE-PROCEED model is most useful for practitioners in planning and developing health promotion [11-15]. However, in China, few of the community based health promotion programs were developed based on health promotion theory, and therefore, their effectiveness were limited.

The central question of health promotion planning is to understand what does the community want, what is actually needed, and, what can actually be done. The three areas overlapped represent what can be accomplished. However, resources, time and other restrictions do not allow everything to be addressed and so areas must be prioritised [16]. In the PRECEDE-PROCEED model, a thorough needs assessment including five phases should be made before planning a health promotion intervention. Currently, needs assessments are not adequately conducted prior to community based health promotion activities in China, resulting in limited impact and poor use of resources. Most CHCs are short of funds and qualified health staff [1]. Health needs assessment can facilitate community participation in health programs, avoid wasting limited resources and provide baseline for program analysis [17]. Considering that the resources available for health care are limited, health needs assessment is one of the key points for successful community based health promotion in China.

Chongqing is located in the upper reaches of the Yangtze River, linking the centre and west of China. Chongqing is one of four municipalities directly under the Central Government. As the newest municipality, Chongqing plays a key role in the development strategy for western China and the Yantze River Three Gorges Dam Project. It covers a large geographical area of 82,000 square km and has over 40 districts or counties, which include 6 urban districts in Chongqing city, 12 poor counties according to national standards and 8 poor counties according to provincial standards. By the end of 2000, the total population was 30. 9045 million, with an urban population of 10.2278 million and a rural population of 20.6767 million [18]. However it lags behind the other municipalities both economically and in terms of social development, for example, per capita GDP and net income per farmer are the lowest rates (5,159 and 1,892 RMB respectively)[18] and life expectancy for urban residents was 76 years [19] and for people from reservoir areas it was 75 years [20]. Recently, in response to ***the National Guidelines for Health Education and Health Promotion *by **MOH of China, the municipal government in Chongqing initiated the program of ***Health Promotion Program for Everybody ***[21], which is a community based health promotion program. However, little is known about the health needs of community members and what community health stations can provide. By using Green and Kreuter's PRECEDE-PROCEED model, this study aims to identify the main health problems having a negative impact on the quality of life of community members, explore the key risk factors associated to a disease or health problem prioritised by the community, and analyze the resources for health promotion in communities in Chongqing city in 2007.

## Methods

### Study setting

We randomly chose two communities in Shapingba (SPB) districts for our study sites: DushiGarden and Tianxingqiao, which represent the communities in Shapingba districts of Chongqing city in terms of economy and population.

### Framework of health needs assessment

According to the PRECEDE-PROCEED model, the needs assessment includes the identification of health problems (Phase 1 and Phase 2), behavioural and environmental risk factors (Phase 3), factors affecting behaviour (Phase 4) and resources in terms of policy and organisations (Phase 5) [16]. In this study we completed the five phases of the assessment to identify community health problems, risk factors and existing resources.

### Triangulation in needs assessment

**Figure 1:**
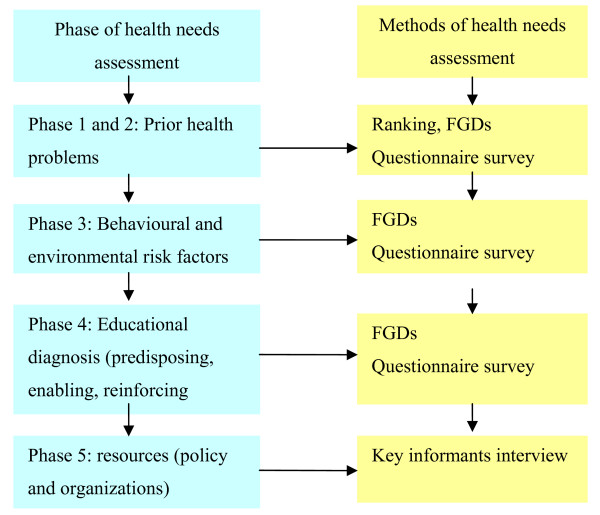
**flow chart of methods in health needs assessment**. The flow chart shows the different methods used in different phases of community health needs assessment.

Triangulation can be used to improve confidence in research findings. Three types of triangulation including data source triangulation, researcher triangulation, and methods triangulation were used in this study (Table 1) [22,23]. Firstly, in data source triangulation we analyzed the main health problems in this district by reviewing existing morbidity data from the Centre of Disease Control (CDC); and investigated health problems and risk factors by ranking, focus group discussions (FGDs) and questionnaire survey. Secondly, triangulation of qualitative and quantitative methods (figure 1) can provide complementary data, which can assure research integrity and rigour [23,24]. Both ranking (a participatory research method) and FGDs(a qualitative research method) were used to identify communities' perceptions of the most important health problems (perceived health needs). Key informants interviews were used to assess health resources for health programs. We conducted a questionnaire survey (quantitative research method) to assess priority health problems ("actual health needs") and risk factors. Thirdly, triangulation of researchers refers to two or more researchers being employed to carry out the same tasks, such as data collection and analysis, which can reduce bias and increases reliability of results [23]. 2 teachers and 3 graduate students carried out and analyzed the FGDs and key informant interviews. The 2 teachers (the first author and the third author) acted as moderators in 4FGDs and interviewers in key informant interviews, 3 graduates (from the School of Public Medicine) acted as moderators in 8 FGDs, 2 teachers independently analyzed data from the FGDs and key informant interviews.

**Table 1:** Triangulation in health needs assessment

**Type of triangulation**		**Example**
Data source triangulation	Existing data review	Analyzed main health problem by reviewing existing health data from CDC
	Investigation	Prioritized health problem by investigating health problem and risk factors with different methods

Methods triangulation	Participation (Ranking)	Prioritized health problem by listing and ranking health concerns (people's perceived needs and priorities)
	FGD	Discussed the health problems, risk factors and their influencing factors (people's perceived needs, priorities, influence factors)
	Key informant interview	Assessed the health resources for health promotion (resources, policy)
	Questionnaires	Surveyed the health problems ("actual needs"), risk factors

Researchers triangulation	Teachers	Role of moderator in 4 FGDs and key informants interviews, and analyzed all FGDs and key informants interviews
	Graduates	Role of moderator in 8 FGDs

### Data collection

#### Ranking

We used the ranking method to identify residents' evaluation of health concerns which have a negative impact on their quality of life. The overview of the process is shown below.

① selecting representative persons---selected eight people who represented the community in terms of sex, age, education and employment; ② generating a list of health concerns --- firstly each participant wrote down their top five health concerns in order of priority, each participant then read out their list and the researcher recorded them on a chalkboard; ③ ranking the list --- using this list each participant ranked their top three items with one, two and three; ④ the scores were added up for each item and the total was divided by the number of participants who ranked each item; the non-ranked items were eliminated and the remaining items were ordered from lowest to highest average rank; when items had the same rank, participants were asked to re-rank their top three concerns from those remaining with one being the highest priority; ⑤ and finally the participants agreed on the priority health concern.

#### Focus group discussions

**Figure 2:**
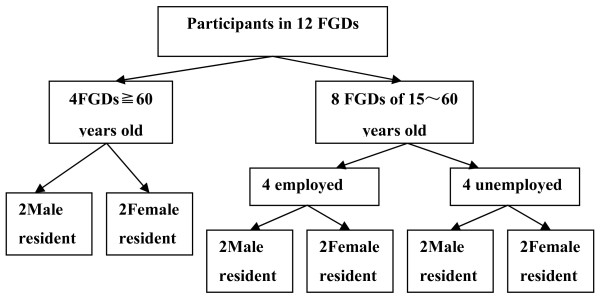
**Schematic view of FGDs distribution by age, gender and employment**. The chart described the distribution of the12 FGDs by age, gender and employment.

Permanent residents of the two communities aged 15 years old and above were selected for the focus group discussions. All participants were recruited with help from the local community committee. At the time of recruitment, the study was explained, including how anonymity and confidentiality would be maintained, prior to obtaining written consent. Twelve FGDs (Figure 2) were facilitated in the local community committee rooms and lasted approximately ninety minutes. Topic guides which used to help facilitate the discussions and they covered questions on community health problems and risk factors. Three members of the research team were present including a moderator, a recorder and an observer, all of whom could speak the local dialect. All FGDs were recorded with the consent of the participants, and terminated when the discussion sufficiently covered the topic and no new information was emerging. At the end of each FGD, a summary of results was read back to the group to enable participants to verify, amend and prioritize emerged issues.

#### Key informants interviews

The leader from the health education department in CDC, 2 directors of the community committees and 4 health workers in the community health stations were purposively selected as key informants. Topic guides were used to explore their perceptions of the current health promotion activities, health promotion policy, organization of health promotion and staff conducting health promotion. All interviews were recorded following consent of the interviewee. Each interview lasted about one hour.

#### Cross-sectional survey

The study population was permanent residents aged 15 years old and above in these communities. Self-administered questionnaires were used to collect data about demographic information, chronic diseases, risk factors (behaviour and lifestyle) and influencing factors of behaviour and lifestyle. There was a response rate of 92.4%: 290 participants were recruited and 268 residents completed the questionnaires.

### Data analysis

Analysis of the FGDs and key informants interviews took place both during and after the research. All recordings were transcribed verbatim. The 'framework approach' was used to analyse the data by two researchers independently. A framework was constructed using the topic guides, checklists and categories emerging from the transcripts, and applied to the data to identify themes [25]. The names of all participants in FGDs and key informant interviews were removed from the quotations in the results to conserve anonymity. The questionnaire data were double entered using Epi Data 6.0. After validation, the data were analyzed by using the Statistical Package for Social Science (SPSS 13.0). Proportion was utilized in a descriptive analysis. Fisher's Exact test or Pearson Chi-Square test was used to compare results between males and females, 60 years old above and 15-60 years, and different educational levels.

### Quality assurance

The research team was trained in the theory and practice of qualitative research and using interactive and participatory methodologies. The topic guides were developed in a participatory way by the research team. All FGDs and key informant interviews were initially analyzed by the first author and then the third author independently analyzed the data again. After discussion, both authors agreed on the findings listed in the results. To ensure trustworthiness, the data relevant to each category were identified and examined using processes of constant comparison [26] and the data were repeatedly re-examined in the light of new emerging themes.

Several methods were employed to guarantee the quality of the questionnaire survey. Each respondent was given written instructions on how to complete the questionnaire. The research team carefully examined the completed questionnaires every day corrected any logical mistakes, and checked the responses with the interviewees. 10 percent of subjects were re-interviewed for quality control. Of these, 95% were closely comparable, showing that the quality of the data collected was good.

### Ethical approval

The local research ethics committees approved the study. Ethical approval was received from the Shapingba DuShi Garden and Tianxingqiao committee and Shaping Ba CDC. Focus group participants gave written consent for their participation after reading a plain language statement describing the study. Survey participants gave oral consent to participation after being read a plain language statement describing the study by the interviewer.

## Results

### Demographic characteristics of study subjects

8 residents participated in the Ranking exercise: 4 females and 4 males; two residents over 60 years old with the rest aged15-60; 4 had junior middle school education level; 4 residents were employed.

Our study recruited 80 participants for 12 FGDs. Both women and men were included. Half of the recruited residents had middle school level education. All of the 28 participants over 60 years old were retired, more than half of participants aged 15 to 60 were unemployed, and the vast majority was married, with only a few individuals being widowed.

As for key informant interviews, we recruited 1 leader (male) from the health education department in CDC, 2 directors (one female and one male) from the community committees and 4 health workers (2 females and 2 males)from the community health stations.

For the survey, 268 residents were recruited. Table 2 provides general information about the study subjects. A high proportion were aged between 15 and 60 years (77.6%); 41.4% of the sample were female and 58.6% were male; the majority of subjects (61.8%) were married; 71.3% had an educational level of primary school or below; almost half (48.6%) were unemployed and 2.0% were students.

**Table 2:** Demographic characteristics of study subjects in questionnaire survey

**Type**		**N**	**%**
Age	15~60	208	77.6
	≥ 60	60	22.4

Sex	male	111	41.4
	female	157	58.6

Marital status	unmarried	36	13.4
	married	197	73.5
	divorced	11	4.1
	Widowed	24	8.9

Education	primary school and below	191	71.3
	middle school	77	13.0

Employment	employed	101	37.7
	student	5	1.9
	retired	16	6.0
	unemployed	146	54.4

### Priority health problems in communities

Phases 1 and 2 involved social and epidemiology assessments to identify priority health problems [16]. In the ranking exercise the residents first listed the following health concerns: cardiovascular diseases (CVDs), diabetes, cancer, kidney diseases (KD), respiratory diseases, gall-stones, gout, hepatitis, leukemia and tuberculosis. In the second round of ranking, they ranked CVDs as their priority health concern, cancer as the second one and KD as the third one (Table 3).

**Table 3:** Results of ranking health concerns*

	**Residents participated ranking**										
				
**Item**	**R 1**	**R2**	**R3**	**R 4**	**R 5**	**R 6**	**R 7**	**R 8**	**Total ranks**	**Number people ranking**	**Average rank**
CVDs	1	1		1		1	1		5	5	1.00
Cancer	2		1	2	1	2	3	1	12	7	1.71
KD	3	3	2		2	3	2		15	6	2.33
Respiratory			3	3	3				9	3	3.00
TB		2						3	5	2	2.5

* "1", "2" and "3" refer to Rank order of the health problems

The questionnaire survey showed that more than 20% of respondents suffered chronic diseases within the last half year; and amongst them the most common illnesses were CVDs (14.1%) (Table 4).

**Table 4:** Chronic diseases reported by residents in questionnaire survey

		**Sex**		**Age**		**Education**	
		
**Diseases**	**%(N)**	**Female** **% (N)**	**Male** **% (N)**	**≧ 60** **% (N)**	**15-60** **% (N)**	**Middle school and above**	**Primary school and below**
CVDs	14.1(38)	9.6(15)	20.7 (23)	25.1(15)	11.1(23)	3.8 (3)	18.3 (35)
Tracheitis	1.5 (4)	2.6(4)	0.0 (0)	6.7(4)	0.0 (0)	0.0 (0)	0.0 (4)
Gout	0.7 (2)	0.0 (0)	1.8 (2)	0.0 (0)	0.9 (2)	0.0 (0)	1.0 (2)
Colpitis	0.7 (2)	1.3(2)	0.0 (0)	0.0 (0)	0.9 (2)	0.0 (0)	1.0 (2)
Pharyngitis	0.7 (2)	0.6 (1)	0.9 (1)	0.0 (0)	0.9 (2)	1.3 (1)	0.1 (1)
Womb tumor	0.7 (2)	1.3(2)	0.0 (0)	0.0 (0)	0.9 (2)	1.3 (1)	0.1 (1)
Bone diseases	0.7 (2)	0.0 (0)	1.8 (2)	0.0 (0)	0.9 (2)	0.0 (0)	1.0 (2)
Diabetes	0.4 (1)	0.0 (0)	0.9 (1)	1.7(1)	0.0 (0)	0.0 (0)	0.1 (1)
Blood stool	0.4 (1)	0.0 (0)	0.9 (1)	1. 7(1)	0.0 (0)	0.0 (0)	0.1 (1)

Total	20.1(54)	15.3(24)	27.0 (30)	35.0 (21)	11.1(33)	6.5 (5)	25.7 (49)

In the FGDs, participants discussed health problems about which they were concerned or from which they suffered. Participants over 60 years old reported that they suffered from a number of diseases associated with old age, with CVDs being the main ones. As for participants aged 15-60, the unemployed described symptoms they had experienced rather than diseases. They included headache, insomnia, tiredness, cough, obesity and gastrectasia. The employed participants were concerned about the stresses and burden of work on their health.

Secondary data collected from the CDC also showed that hypertension, coronary heart disease and stroke (all of which are CVDs) are the primary causes of death in this area.

Table 5 summarises the results of all research methods and resources.

**Table 5:** Priority of health needs in study place assessed by different resources *

**Data source**	**CVDs**	**Respiratory**	**Tracheitis**	**Cancer**	**DK**	**Gout**
CDC	1	2	3			
Ranking	1			2	3	
FGDs	1					
Questionnaires	1		2			3

* "1", "2" and "3" refer to Rank order of the health problems

Table 6 shows that male residents, residents over 60 years old and residents of low education level were more susceptible to CVDs.

**Table 6:** Population distribution of CVDs in residents from questionnaire survey

**Type**		**N**	***X*^2 ^value**	**P value**
sex	female	15	6.663	0.010
	male	23		
age	≧ 60	15	7.439	0.006
	15-60	23		
education	middle school and above	3	8.240	0.04
	primary school and below	35		

### Behaviour and environment diagnosis and educational diagnosis

Phase 3 of the needs assessment focused on the identification of the determinants of the health problem. Phase 4 involved the identification, sorting and classification of the 'predisposing, enabling and reinforcing factors' that affect the health-related behaviours [16].

According to recent literature, the modifiable risk factors of CVDs mainly include cigarette smoking, physical inactivity, eating food high in salt and sugar, eating fatty food and few vegetables [27,28]. The questionnaire survey included questions on the behaviour and lifestyle of the respondents (Table 7). The findings showed: 12.7% of respondents reported smoking behaviour; 73.5% were physically inactive; 40.1% had a preference for fatty food; 46.5% liked to eat salty food; and 40.5% ate sugary food. *X*^2 ^test showed that males, residents over 60 years old and people who had fewer years of education tended to have more risk factors for CVDs. Residents did not report any environmental pollution.

**Table 7:** Health risk factors of CVDs reported by residents in questionnaire survey^⋆^

		**Sex**		**age**		**education**	
		
**Risk factors**	**Total** **N (%)**	**Female** **N (%)**	**Male** **N (%)**	**≧ 60** **N (%)**	**15-60** **N (%)**	**Primary school and below** **N (%)**	**Middle school and above N (%)**
smoking	34 (12.7)	1(0.1)	33(29.7)**	2 (3.3)	32(15.4)*	21(10.9)	13(16.9)
Physical inactive	197 (73.5)	90(57.3)	107(96.4)**	58(96.7)	13(66.8)**	163(85.3)	34(44.2) **
high-fat foods	108 (40.3)	30(19.1)	78(70.3)**	33.3 (20)	88(42.3)	97(50.8)	11(14.3) **
salty foods	125 (46.6)	85(54.1)	40(36.0)*	54(90.0)	71(34.1)**	98(51.3)	27(35.1) *
sugary foods	109 (40.7)	35(22.3)	74(66.7)**	50(83.3)	59(28.3)**	90(47.1)	19(24.7) **
Lack of vegetables	34 (12.7)	18(11.5)	17(15.3)	15(25.0)	19(9.1)**	24 (12.6)	10(12.9)

^⋆ ^χ^2 ^test used to compare between males and females, lower than 60 years and older than 60 years, and educational levels.

*P ≦ 0.05

**P ≦ 0.01

The survey showed that only a few people could name some risk factors for CVDs, with 73.50% not knowing any risk factors (Table 8). When asked about their health education needs, 33.5% of respondents perceived a need for health education in order to know the risk factors for CVDs and preventive measures, 19.1% wanted guidance about diet and 6.4% wanted a medical consultation (Table 9). However, more than half the respondents (55.97%), and in particular respondents with primary school education or below, perceived that they had no health education needs. In addition, more than 50% respondents had never received any health promotion (Table 10).

**Table 8:** knowledge about CVDs of residents in questionnaire survey^⋆^

		**Sex**		**age**		**education**	
		
**Risk factors**	**N (%)**	**Female** **N (%)**	**Male** **N (%)**	**≧ 60** **N (%)**	**15-60** **N (%)**	**primary school ** **and below**N (%)	**Middle school ** **and above N (%)**
Unhealthy diet	28 (10.4)	17 (10.8)	11 (9.9)	15(25.0)	13 (6.3) **	10(5.2)	18 (23.4) **
physical inactivity	22 (8.2)	10 (6.4)	12 (10.8)	8 (13.3)	14 (6.7)^※^	8(4.2)	14 (18.2) **
smoking	15 (5.6)	10 (6.4)	5 (4. 5)	8 (13.3)	7 (3.4)^※^**	6(3.1)	9 (11.7)^※^*
stress	16 (5.9)	6 (3.8)	10 (9.0)	5 (8.33)	11 (5.3)^※^	7(3.7)	8(10.4)^※^
Don't know	197(73.5)	114(72.6)	83(74.8)	24(40.0)	173(83.2) **	145(75.9)	52(67.5)

^⋆^χ^2 ^test used to Compared between male to female, lower than 60 years and older than 60 years, and educational differences

^※ ^Fisher's Exact

*P ≦ 0.05

**P ≦ 0.01

**Table 9:** Perceived needs reported by residents in questionnaire survey^⋆^

		**Sex**		A**ge**		**Education**	
		
**Perceived needs**	**N (%)**	**Female** **N (%)**	**Male** **N (%)**	**≧ 60** **N (%)**	**15-60** **N (%)**	**Primary school ** **and below N (%)**	**Middle school ** **and above N (%)**
health knowledge	84(33.5)	54(34.4)	30(27.0)	50(83.33)	34 (16.4) **	30(15.7)	54 (70.1) **
guidance of diet	48(19.1)	32(20.4)	16 (14.4)	23 (38.3)	25 (12.0) **	20(10.5)	28 (36.4) **
health consultation	16 (6.4)	9 (5.7)	7 (63.1)	6 (10.0)	10 (4.8)	9(4.7)	7 (9.1)
No needs	150(55.9)	90(57.3)	60(54.1)	34 (56.7)	116 (55.8)	123(64.4)	27 (35.1) **

^⋆^χ^2 ^test used to Compared between male to female, lower than 60 years and older than 60 years, and educational differences

**P ≦ 0.01

**Table 10:** Accessibility to community health promotion activities reported by residents in questionnaire survey^⋆^

		**Sex**		**Age**		**Education**	
		
**Accessibility**	**N (%)**	**Female** **N (%)**	**Male** **N (%)**	**≧ 60** **N (%)**	**15-60** **N (%)**	**Primary school ** **and below N (%)**	**Middle school ** **and above N(%)**
Never participating	109(40.9)	76(48.4)	33 (58.6) **	45(75.0)	64 (30.8) **	57(29.8)	52 (67.5) **
Health information propaganda	100(37.3)	65 (41.4)	35 (31.5)	15(25.0)	85 (40.9)*	70(36.4)	30 (38.9)
guiding of life style	68(25.4)	30 (19.1)	38 (34.2)*	5 (8.3)	63 (30.3) **	38(19.9)	30 (38.9) **
Training of quitting smoking or drinking	6(2.8)	1 (0.1)	5 (0.5)^※^*	3 (5.0)	3(1.4)^※^	2 (1.0)	4 (5.2)^※^*

^⋆^χ^2 ^test used to Compared between male to female, lower than 60 years and older than 60 years, and educational differences

^※ ^Fisher's Exact

*P ≦ 0.05

**P ≦ 0.01

In the FGDs, participants discussed their lifestyle and factors influencing their behaviour. Only the female participants perceived that there was widespread unhealthy behaviour within the communities. They explained that many adults drank beer every night; many adults did not do a physical exercise; and children preferred to eat junk food or food they thought to cause cancer such as barbeque food. They associated CVDs with this unhealthy lifestyle. Although the minority of FGD participants had some knowledge about risk factors for CVDs such as smoking and physical inactivity, most participants agreed that they didn't know that their diet put them at risk of CVDs and did not know how to eat healthily. Most participants of the FGDs recognised that they needed some information about healthy lifestyle and guidance on diet. However, the young male employed residents perceived that they had no need for information. Participants also described that they had little access to health education activities and that leaders in workplaces and schools placed little importance on these activities. Some participants also reported that the cultural norm of playing Majiang encourages a sedentary lifestyle (Table 11).

**Table 11:** Results of behaviour and environment diagnosis and educational diagnosis in FGDs

**Factors**	**Results**	**Quotation**
Behavior and environment diagnosis	Only female participants emphasized the unhealthy lifestyle everywhere in the community, associated CVDS with that unhealthy lifestyle	*"Yes, I agreed with them, I know that hypertension, cardiovascular diseases are very common around me, I think those people have unhealthy lifestyle, for example, they sleep just after eating, and they seldom participated any physical activities"*. *"I often see the children taking the junk food, therefore those children become obese, and I worried their health very much..."*

Predisposing factors	The minority described some risk factors and preventive measures. The majority didn't know how to behaviour healthily and perceived a need for health information guidance about diet Young male employed residents identified no need for health education	*"We don't know how to eat and how to behave, we often play Majiang with friends, otherwise we don't know what to do for fun."* *"For the elderly, if you can give some information about the common diseases like hypertension and CVDs, this would be helpful...."* "*I think healthy lifestyle should be cultivated from childhood. It is very important. Now you can see many young obese children. So I think you should tell us how to have a healthy lifestyle. This knowledge should be in a textbook for children and there should be school health education."* *"We are healthy, we rarely get a cold. We don't know what health needs we have, we don't worry about this"*

Enabling factors	Poor accessibility to health knowledge Less attention to health promotion from leaders in work places and primary schools	*"Sometimes, there is some health education, but it is provided by a company. They usually give some information about some diseases, and then they introduce their products and persuade you to buy them. It's just a way for them to advertise their products. So we don't like this so-called health education."* *"We are busy in the workplace during the day, so it is difficult for us to participate in health education in the community. So it is better to hold health education in the workplace. The policy says this, but our leader pays no attention to health education."* *"...I hope the school teachers spend some time teaching about healthy lifestyle, but it seems that they never consider this."*

Reinforcing factors	Local people like playing Majiang, it is part of the local culture	*"In Chongqing, you can see Majiang pubs everywhere; playing Majiang is a one way to have fun, all of our friends like it."*

### Policy and administration diagnosis

This phase required an analysis of the policies, resources, and circumstances in an organizational setting and context that either hinder or facilitate the development of a health project [16].

The CDC director described several national and local policies and programs that have influenced the development of health education. He reported that the national policy for health promotion was available in the CDC. He described that *the National Guidelines for Health Education and Health Promotion* by the Ministry of Health of China(2005-2010) in 2005, and the *National health promotion project for Chinese farmers *launched by central government in 1994, have helped develop health promotion in China in the last 10 years. He further reported that the central government made several policies to improve this program, which included *Decision on health reform and development by Chinese Communist party and the State Council, Guidance on urban community health service development *and *Decision on further strengthening urban community health service development *made in 1997, 2001 and 2002 respectively. He also mentioned that in 2006 the Chongqing local government sponsored a program called *Health Promotion Program for Everybody *in response to national policies and programs. He emphasized that health education departments were created in CDCs. Although, health education is one of the six functions of the community health station, health workers have many other responsibilities. Key Informants in the community health stations and community committees reported that the implementation of health promotion lagged behind the national and local policy mainly because of a lack of staff. Health promotion activities were not carried out on a regular basis, but some health education was given to communities on "health days" such as World Tuberculosis Day. In addition, school health promotion activities were seldom carried out for students. For example:

"...there are policies from central government to local government on health promotion, eg. the National Health Promotion Project for Millions of Chinese Farmers. In Chongqing, we have health promotion for everybody. In CDC we set up a health education department, and assigned professional persons to it. They are responsible for guiding health education in this district. In the community health station, the health workers carry out community health education activities as part of their job" (Director from CDC)

"...in our community health education isn't organized often. There are some health education activities on March 24-tuberculosis education day. I don't know about health promotion in schools, but it seems that few activities are organized."(Director in community committee)

## Discussion

### Role of community health needs assessment

During the last few decades, attention has been paid to the role of community health services in achieving large-scale changes in behaviour. A number of health promotion initiatives have used a community approach for the prevention and control of chronic diseases [29]. Successful community health promotion requires a thorough understanding of community health needs, active participation of community members, use of existing community resources, involvement of all relevant local constituencies and incorporation of multiple intervention strategies [17]. Health needs assessment describes health problems of a population and determines priorities for the most effective use of resources by utilizing epidemiological, qualitative and comparative methods. It ensures that the health service uses its resources to improve the health of the population in the most efficient way [30]. In carrying out a community needs assessment with active community participation, stakeholders of future health programs are involved. This involvement helps community members and decision-makers understand the health problems within their communities and identify strategies for the health program. Community participation and local-level policy making play important roles in facilitating access to, trust and implementation of potential health program [31-36]. Therefore, we conducted a needs assessment using the PRECEDE-PROCEED model as a conceptual framework in order to provide a foundation for communities to plan health promotion targeted at the priority health problem.

### CVDs - priority community health needs

Our study showed that CVDs were the priority health concerns, particularly among males aged above 60. CVDs include coronary heart disease (CHD), cerebrovascular disease, hypertension, heart failure and rheumatic heart disease [37]. CVDs are the most common causes of death in Western societies [38]. CVDs in low and middle income countries account for 85% of the global CVDs disease burden [39] and by 2001 had become the leading cause of death in developing countries [40,41]. The mortality rate of CVDs and prevalence of major CVDs risk factors has substantially increased in China [42,43]. Both the Chinese fourth national health survey in 2008[44] and the Chinese third national retrospective survey on causes of death in 2007[45] also showed CVDs to be the main health problems and the leading causes of death in China, and their prevalence were four to five times of that in the developed countries. Among the first 5 leading causes of death in Chongqing were respiratory diseases, cancer, cerebrovascular diseases, heart attack, injury and poisoning [45]. Two studies demonstrated similar results in Chongqing [19,46]. When setting health priorities in a community, the preventability of the health problem, the number of people potentially and actually affected by the problem, severity and public concern should be taken into account [47]. This study together with previous reports confirmed that CVDs are important causes of morbidity and mortality and should be the priority health problem in the study communities.

### Unhealthy behaviours, lifestyle, and work stress associated with CVDs

Although there are a number of new risk factors found to be associated with CVDs, the traditional risk factors play the most important role in incidence and mortality of CVDs. The key modifiable traditional risk factors are "lifestyle" [48] including smoking, physical inactivity, diet and obesity [27]. Unhealthy eating is one of the important components of lifestyle that contributes to the development of diseases such as cardiovascular diseases [49]. Despite some improvements, diets still contain too much saturated fat, sugar and salt and insufficient vegetables, fruits and fish [50]. This study identified several CVDs risk factors in the community members (Table 7). We found a high proportion of respondents had unhealthy dietary behaviours, and in particular 43.09% ate food high in salt and sugar which is more than has previously been reported (21.19%) [46]. The majority (73.5%) of respondents were physically inactive, which is similar to the findings of the fourth national health survey where 23.5% participated in physical activity. However other studies showed a range of findings, with a lower proportion (62.32%) and higher proportion (82.70%) of physical inactivity in their study populations [45,46]. Our study further highlighted that males and residents with fewer years of education showed more risk factors for CVDs. Literature supports that males and people with fewer years of education were more susceptible to CVDs [51-53]. Therefore, we can deduce that men and residents with fewer years of education are more likely to be at high risk of CVDs in this study site. It is clear that changing unhealthy behaviours and lifestyle is an important measure to prevent and control CVDs in Chongqing cities, particularly among men and people with fewer years' education.

An important problem was raised by the employed residents. They were concerned that stress caused by their work affected their health. Chandola et al. reported that work stress can directly affect CHD and indirectly impact on it by affecting behaviour such as low physical activity and poor diet [54]. Therefore, stress caused by work should be considered as a risk factor for CVDs in this study population.

### Addressing predisposing, enabling, and reinforcing factors

The best way to design programs to achieve positive changes in health behaviour is to understand why people behave as they do and what might motivate them to change [48]. According to Green et al. [16], reasons for behaviour can be classified into predisposing, enabling and reinforcing factors, and then more responsive intervention strategies for health promotion can be developed for each factor. In this study, "educational diagnosis" explored those three factors.

Predisposing factors are the individuals' or populations' knowledge, attitudes, beliefs, values and perceptions that facilitate or inhibit health behaviours [16]. Both FGDs and questionnaires consistently identified that the majority of residents lack knowledge about risk factors for CVDs. Although some people expressed a need for information about health and diet, more than half reported no needs. In the FGDs, residents believed that they were not at risk of CVDs. This belief may explain why they perceived no need for health education. Perceptions of being at high risk of developing a disease and knowledge of the disease are known to influence motivation for behaviour change [16,49]. Therefore, in this study, community members' beliefs, perception of needs and limited knowledge may affect their willingness and ability to make behaviour changes.

Enabling factors are the requisite skills, environment and resources required to perform health behaviour [16]. Health education and promotion activities are environmental factors. Actors in policy making, health education and health promotion play important roles in shaping those environmental factors [49]. In this study, almost half the respondents have little access to health promotion activities. Their perceptions of the current health education in the community as being primarily for companies to advertise their products influenced their enthusiasm to participate in future health promotion activities. Previous studies reported that work site health promotion has been used to reduce the risk of CVDs [55] and health promotion in schools helped the school population with achieving healthy lifestyles and supporting healthy behaviours by developing supportive environments [56,57]. However, our study identified perceptions that health promotion in the workplace and schools was not important as well as few or no activities happening in these places. Therefore, low accessibility to community health promotion, the poor quality of current health promotion, and lack of work site or school health promotion acted as negative enabling factors.

Reinforcing factors are those consequences of action that determine whether the action is supported positively or negatively [16]. Majiang was a game created in China long ago and plays an important role in traditional Chinese culture. It is played with four persons seated around a table at home or a Majiang pub in the community. Our study found that "the Majiang culture" is very popular. Although there may be benefits to playing Majiang such as making friends, engaging in business transactions, relaxation and entertainment, the game require the players to sit for long periods and may be harmful for their health. Residents may take part in this because of a lack of alternative entertainment or recreation. Therefore, the popular Majiang culture acts as a reinforcing factor supporting the sedentary life of residents. This should be taken into account in setting up intervention strategies.

In conclusion, we identified three categories of factors as follows: predisposing factors included residents' knowledge, health awareness and belief and perceived health needs. Accessibility to health promotion, attention to health promotion from leaders in work places or primary schools and health resources (health promoters, policy, organization) were enabling factors. Socio-cultural factors were reinforcing factors.

All the risk factors for CVDs are modifiable through simple behaviour and lifestyle changes. To address the negative predisposing, enabling, and reinforcing factors described above in order to modify the risk behaviour and lifestyle for CVDs, the following strategies can be considered: to plan community based health education; to provide and improve residents' accessibility to work site, school and community health promotion; to provide information about health including enhancing life skills which will increase the options available to people to exercise more control over their own health and over their environments, and to make choices conducive to health; and to advocate more healthy recreation activities in the community such as setting up a sports ground.

### Strengthening policy implementation, organization and manpower

The Ottawa Charter outlines five areas for health promotion action. The first one is to build healthy public policy [58]. Policy and administration diagnosis showed that the national and municipal policies for community health promotion were available, but those policies were not being implemented at the community level. There is a need for strategies to be developed at the local level to implement the policies. For example, a policy to strengthen the role of the community health service stations in health promotion should be enacted and a community policy on school and worksite heath promotion should be developed. In addition, a healthy food policy and a law that prohibits the selling of tobacco products to youths under 18 years of age should be considered. Organizations for health promotion do exist in the CDC and community. However it is necessary to establish health promotion committees with representatives from the municipal, provincial, county, district and street levels. These committees are responsible for facilitating the development, implementation and management of health promotion projects [59].

Shortage of health promotion professionals can hinder the implementation of health promotion projects. Therefore, training and developing existing community health staff or identifying and training voluntary health promoters can be strategies to address this problem. Other strategies include: mobilising the communities and leaders within the communities to organise and fund health promotion programs; and establishing community information networks.

### Limitations

Although we utilized various ways of triangulation in this study in order to increase the reliability of the findings, the small number of FGDs and interviews may limit the generalisation of the findings. This study identified CVDs as the most important health problem for the study communities. While the national survey showed CVDs are ranked third in the top five causes of mortality in Chongqing. This difference may be due to the different study samples: this study represented the 6 districts in Chongqing city, whereas the national survey included the 40 districts and counties of Chongqing. It may also be due to the different methods and data sources. The national survey used data from records in health facilities with interviews to confirm these results and a household survey. However, this study used records from CDC, survey, FGDs and ranking exercise. In addition, the sample size for this study was small. We need a further study with a larger sample size in order to examine this difference. A future study should use mixed research methods to explore in more detail the health promotion resources and policies in the community.

## Conclusion

A health program cannot be based upon what we, as professionals, decide the public does or does not know about a specific issue [60]. Planning, key to any successful health promotion project, must include a thorough needs assessment using literature reviews and investigations. The main evidence in this paper is that CVDs were identified as the priority health problems in communities; risk factors mainly included behaviours and lifestyle (dietary behaviours), which were particularly evident in men and people with fewer years education; residents' limited knowledge, beliefs, perceived needs, low accessibility to health promotion and culture played key roles in affecting residents' behaviour and lifestyle; there were problems in the implementation of policy and organization for health promotion. The results suggest that health project planning should target CVDs and their risk factors. Strategies for health promotion should take these factors into consideration. Policy, organization and manpower need further strengthening.

## Competing interests

The authors declare that they have no competing interests.

## Authors' contributions

Jia Cao, Jia He and Yang Wang were responsible for the study design and coordination of the research project. Ying Li, Daikun Li and LinHui performed the data collection, management and analysis. All authors participated in interpretation of the findings. YingLi drafted the manuscript. All authors read and approved the final version of the paper. All authors confirm that the manuscript has not been published in any journal and other citable form.

## Pre-publication history

The pre-publication history for this paper can be accessed here:


